# Effect of direct oral anticoagulants in patients with atrial fibrillation with mitral or aortic stenosis: A review

**DOI:** 10.3389/fcvm.2022.1070806

**Published:** 2022-11-16

**Authors:** Guigao Guo, Shucheng Liang, Zeyu Guan, Ke Zhu

**Affiliations:** ^1^Department of Cardiovascular, Zhuzhou Hospital Affiliated to Xiangya School of Medicine, Central South University, Zhuzhou, China; ^2^Faculty of Medicine, Macau University of Science and Technology, Macau, Macau SAR, China

**Keywords:** direct oral anticoagulants, warfarin, atrial fibrillation, mitral stenosis, aortic stenosis

## Abstract

**Background:**

Several studies have summarized the clinical performance of direct oral anticoagulants (DOACs) in atrial fibrillation (AF) patients with mitral stenosis or aortic stenosis. The significance of this review was to provide clinicians the latest update of the clinical application of DOACs in managing this specific population.

**Methods:**

Literatures from the PubMed database up to July 2022 were screened for inclusion. Studies on the effect of DOACs in patients suffering from AF with mitral or aortic stenosis were assessed for further selection.

**Results:**

Results from four studies were gathered: the RISE MS trial, the DAVID-MS study, and two observational studies. In the Korean observational study with a 27-month follow-up duration and a sample population consisted of patients with mitral stenosis and AF, the thromboembolic events happened at a rate of 2.22%/ year in the DOAC group and 4.19%/year in the warfarin group (adjusted hazard ratio: 0.28; 95% CI: 0.18–0.45). Intracranial hemorrhage occurred at rates of 0.49% and 0.93% in the DOAC and the warfarin groups, respectively (adjusted hazard ratio: 0.53; 95% CI: 0.22–1.26). In the Danish observational study, which had a sample pool with AF patients with aortic stenosis, reported that the adjusted hazard ratios for thromboembolism and major bleeding were 1.62 (95% CI, 1.08–2.45) and 0.73 (95% CI, 0.59–0.91) for DOACs compared with warfarin during 3 years of follow-up. In the RISE-MS trial involving AF patients with mitral stenosis, there were no differences in ischemic stroke, systemic embolic events, or major bleeding between the rivaroxaban vs. warfarin groups during a 1-year follow-up as well as equal rate of increased thrombogenicity in the left atrial appendage at 6 months. The rate of silent cerebral ischemia at 12 months was higher in the warfarin group (17.6%) than that in the rivaroxaban group (13.3%).

**Conclusions:**

Current published studies supported DOACs' effectiveness in preventing thromboembolism in patients of AF with mitral or aortic stenosis. Further clinical trials could confirm these findings.

## Introduction

Valvular heart disease (VHD) has a rising prevalence in the elderly population over 75 years old (1). Among the moderate-to-severe VHDs, mitral or aortic stenosis happen with rates of 11 and 9%, respectively. Mitral stenosis (MS) is the most common valve stenosis, characterized by the narrowing of the mitral valve, which is crucial to prevent backflow from the left ventricle, followed by the occurrence of life-threatening complications such as atrial fibrillation (AF) and heart failure (2). Aortic stenosis (AS) is featured by the narrowing of the aortic valve which subsequently restricts the ejection of blood from the left ventricle, leading to high ventricular pressure and serious complications like AF (3). It has been shown that patients develop AF associated with MS and AS in a rate of 66.6% (4) and >9% (5) respectively, of which 3–7.5% of the patients are complicated by thromboembolic stroke.

Current guidelines of anticoagulation for AF in patients with non-valvular heart disease recommend that warfarin, a vitamin-K-dependent anticoagulant (VKA), is the drug of choice (6–8). However, such guidelines do not include AF combined with VHDs like mitral or aortic stenosis, which leaves patients developing both VHDs and AF with less therapeutic options beyond traditional warfarin administration. There is an urgent need for the establishment of a more inclusive guideline that provides alternative anticoagulation involving the usage of direct oral anticoagulants (DOACs) for patients with both VHD and AF (9). More recent studies have shown that DOACs are superior to warfarin for the prevention of systemic embolism in patients with AF (10–14), and even have a significant reduction in intracranial hemorrhage (12, 15–19). The better effect of DOACs compared with warfarin is also found in the AF specific population (20–24) and is well supported by cohort studies (25–28). However, only a few have specifically studied the efficacy and safety outcomes of DOACs compared with warfarin in AF patients with MS or AS (29–33). In this review, we discussed all the relevant studies regarding the effect of DOACs in AF patients with MS or AS.

## Methods and results

Two investigators conducted independent searches on online database. Combinations of the following keywords were used to generate a search for relevant articles on the PubMed database up to July 2022: dabigatran, rivaroxaban, apixaban, edoxaban, direct oral anticoagulants, novel anticoagulants, DOAC, NOAC, warfarin, atrial fibrillation, mitral stenosis, aortic stenosis, and valvular heart disease. Observational studies or randomized controlled trials (RCTs) were selected if they satisfied the following criteria: AF patients with mitral or aortic stenosis treated with DOACs compared with warfarin.

**Figure 1:**
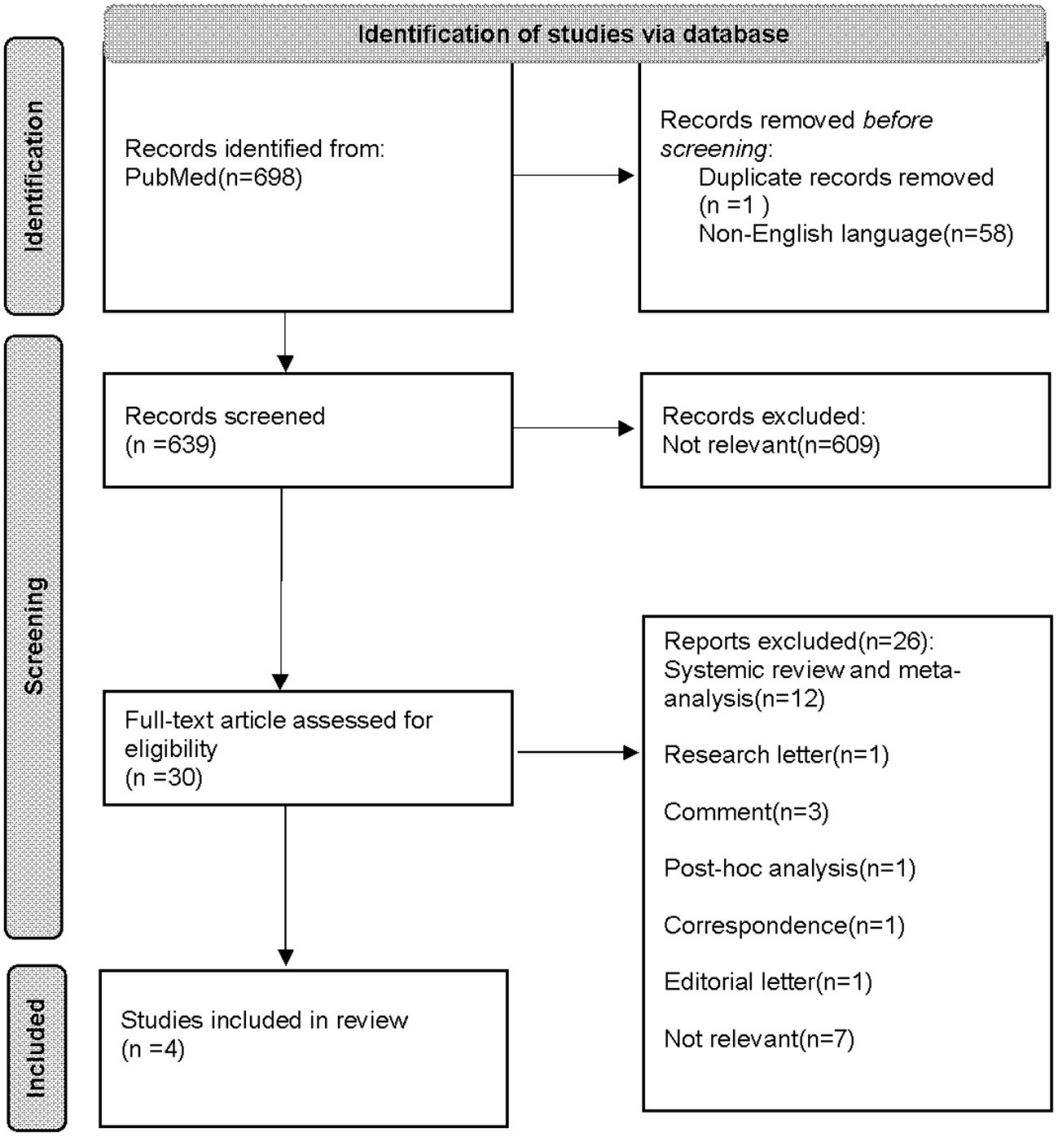
PRISMA flow diagram that summarizes the literature search process.

A total of 698 articles were identified from the database for initial screening, 30 of which met the inclusion criteria and were retrieved for full-text article reading. Upon assessment for eligibility, 19 articles were excluded for not being either a RCT or observational study, seven articles were removed due to irrelevance. Only four articles eventually out of 30 matched the criteria and were included in this review. Among the four articles reviewed, two are observational in design, both are multicenter retrospective cohort studies, one using 1 to 1 propensity score matching, the other one using target trial emulation. The rest two studies are RCTs, one of which is still a protocol, with results not yet available. The whole search and selection process is summarized in Figure 1. The study design and baseline information of the studies are demonstrated in Table 1.

**Table 1:** The baseline data of the included studies in this review.

**References**	**Study treatment**	**Study design**	**Baseline characteristics of the population**	**Efficacy outcome results**	**Safety outcome results**
Kim et al. (33)	Apixaban (n = 192), dabigatran (n = 367), rivaroxaban (n = 472), or edoxaban (n = 84) vs. Warfarin (n = 1,115), dosage unmentioned	Multicentre, retrospective cohort study; 1 to 1 propensity score matching	Age: DOAC 69.2 vs. warfarin 70.2 years; Hypertension: DOAC 1076 vs. Warfarin 2080; previous stroke: DOAC 518 vs. Warfarin 521; mean CHA DS 2 2-VASc score = 5.2	Stroke or systemic embolism: DOAC 2.22%/year (*n =* 30) vs. Warfarin 4.19%/year (*n =* 146)	Intracranial hemorrhage: DOAC 0.49%/year (*n =* 7) vs. Warfarin 0.93%/year (*n =* 36)
Sadeghipour et al. (29)	Rivaroxaban 20 mg/day or 15 mg/day (CrCl <50ml/min; *n =* 20) vs. Warfarin with target INR 2-3 (*n =* 20). Study discontinued due to concerns raised by COVID-19.	Single center, open-labeled, parallel-group, pilot registered RCT (RISE MS)	Age: Rivaroxaban 60 vs. Warfarin 56 years; BMI: Rivaroxaban 27.1 vs. Warfarin 27.8 kg/m2 Hypertension: Rivaroxaban 5(25%) vs. Warfarin 4 (20%) HAS-BLED score: Rivaroxaban 0 vs. Warfarin 0	Stroke or systemic embolism: DOAC (n = 0) vs. Warfarin (*n* = 0)	Major bleeding: Rivaroxaban (*n =* 0) vs. Warfarin (*n =* 0); Clinically nonmajor bleeding: Rivaroxaban (*n =* 1) vs. Warfarin (*n =* 0)
Melgaard et al. (30)	Apixaban (*n =* 1105), Dabigatran (*n =* 323), edoxaban (*n =* 38) or rivaroxaban (*n =* 891) vs. Warfarin (*n =* 1369)	Multicenter, retrospective “target trial” emulation	Median age: NOAC 82 vs. Warfarin 79 years; Previous aortic valve intervention: NOAC 497 (21.1%) vs. Warfarin 432 (31.6%); Hypertension: NOAC 1616 (68.6%) vs. Warfarin 957 (69.9%); Heart failure: NOAC 1008 (42.8%) vs. Warfarin 670 (48.9%)	Thromboembolism: Per protocol analysis: Warfarin (n = 19) vs. NOAC (n = 62); Intention-To-Treat analysis: Warfarin (n = 36) vs. NOAC (n = 77);	Major bleeding: Per protocol analysis: Warfarin (*n =* 119) vs. NOAC (*n =* 163) Intention-To-Treat analysis: Warfarin (*n =* 171) vs. NOAC (*n =* 184)
Zhou et al. (31)	Dabigatran 110/150 mg BD (*n =* 343) vs. Warfarin (*n =* 343; INR 2-3 and TTR > 65%) protocol	Protocol, randomized, open-label study (DAVID-MS)	N/A	Stroke or systemic embolism	Ischemic stroke, systemic embolism, hemorrhagic stroke, intracranial hemorrhage, major bleeding and death

N/A, not applicable.

Among four studies included in this review (29–31, 33), the primary outcomes and safety outcomes are summarized in Table 1. The Korean observational study by Kim et al. (33) included 2,230 AF patients with MS, of which 30.6% were males. It was reported that thromboembolic events occurred at a rate of 2.22%/year in the DOAC group and 4.19%/year in the warfarin group (adjusted hazard ratio for DOACs vs. warfarin: 0.28; 95% CI: 0.18–0.45), while intracranial hemorrhage occurred in 0.49% in DOACs group and 0.93% in warfarin group (adjusted hazard ratio for DOACs vs. warfarin: 0.53; 95% CI: 0.22–1.26). The incidence rates of all-cause death were 3.45%/year in the DOAC arm and 8.08%/year in the warfarin arm. The overall survival curve showed a lower all-cause death in the DOAC group compared with the warfarin group. The estimated 3-year major bleeding-free survival was 87.6% for DOACs and 83.6% for warfarin. In the RISE-MS pilot RCT (29), 37 patients with AF and MS were recruited and randomized into either rivaroxaban (*n* = 18) or warfarin (*n* = 19) groups. This study reported no symptomatic ischemic stroke or systemic embolic events during the 1-year follow-up. For the safety outcomes, there was no major bleeding in neither group, but 1 clinically relevant nonmajor bleeding in the rivaroxaban group which was explained by increased menstrual bleeding. For exploratory outcomes, the rates of increased thrombogenicity in the left atrial appendage (LAA) assessed by transesophageal echocardiography (TEE) at 6 months and silent cerebral ischemia at 12 months assessed by brain magnetic resonance imaging (MRI) were explored. There were 11 patients in each group agreed to undergo the TEE assessment. There were 15 patients in the rivaroxaban group and 17 patients in the warfarin group accepted the MRI assessment. As results, both groups reported 27.2% rates of increased LAA thrombogenicity, whereas the rates of silent cerebral ischemia were 13.3 and 17.6% in the rivaroxaban and warfarin groups, respectively. Zhou et al. (31) published the protocol of the DAVID-MS trial on the effect of dabigatran vs. warfarin in patients with AF and MS.

The Danish observational study by Melgaard et al. (30) included 3,726 patients with AF and AS who had been prescribed for either a DOAC (*n* = 2,357) or warfarin (*n* = 1,369). During a median follow-up 14 months, the adjusted hazard ratio for thromboembolism was 1.62 (95% CI, 1.08–2.45) for DOACs compared with warfarin. The estimated 3-year thromboembolic-free survival was 94% in the DOACs group and 96% for the warfarin group. For the safety outcomes, the adjusted hazard ratio for major bleeding was 0.73 (95% CI, 0.59–0.91) for DOACs compared with warfarin.

## Discussion

One of the earliest studies looking into the effect of DOACs in patients with MS was conducted by Kim et al. (33) in 2019. This study presented a retrospective analysis to validate the effectiveness and safety outcomes of DOACs vs. warfarin in patients with MS. Patients were selected using the Korean health insurance database between 2008 and 2017 that were identified with AF and MS. A total of 2,230 patients were enrolled with matching baseline characteristics and 1:1 propensity score matching, of which patients in the DOACs or warfarin group were divided evenly. The primary outcomes of interest were ischemic stroke and systemic embolism over a follow-up of 27 months, and the safety outcomes were intracranial hemorrhage and all-cause death over the same course of follow-up. Thromboembolic events happened in rates of 4.19% per year and 2.22% per year and intracranial bleeding occurred in rates of 0.93% per year and 0.49% per year in warfarin and DOACs groups, respectively. Although the results seemed to support that DOACs were more effective and safer than warfarin, since the use of DOACs was off-label administered, it was difficult to overcome the confounding factors given a narrow range of baseline characteristics. Moreover, comparing to RCTs, observational retrospective studies have less restrictions as well as less consistency in terms of experimental design due to the fact that the data gathered was collected from different healthcare providers. Such characteristics of all observational analysis make them prone to selection bias. Therefore, results from such observational study should be interpreted critically and the data should only be used for “hypothesis-generating”.

In another observational study conducted in 2021, Melgaard et al. (30) collected data from Danish nationwide registries between 2013 and 2018 with the intent to compare effectiveness and safety of DOACs with warfarin in patients with AF and AS. Similar to the situation regarding treatment for patients with both AF and MS, there is lack of information and update about the guidelines on the usage of DOACs for patients carrying AF and AS. Melgaard et al. has highlighted the necessity of exploring the efficacy of DOACs for such indication in the observational study. A total of 3,726 patients with AF and AS satisfied selection criteria, in which 2,357 patients initiated DOACs and 1,369 patients used warfarin. Throughout 3 years of follow-up, thromboembolism happened in a rate of 3.3% in the DOAC group and 2.6% in the warfarin group, indicating a higher risk of thromboembolism in treatment with DOACs, whereas major bleeding occurred in a rate of 13% and 7.8% in the DOACs and warfarin groups, respectively. A major drawback of this study is its non-randomized design, which is common in every observational study as discussed above, making confounding factors unavoidable. Another limitation is that even though the comparison is between DOACs and warfarin, the study did not specifically compare two single drugs. Instead, patients prescribed with apixaban, dabigatran, edoxaban, and rivaroxaban were all counted into analysis, which potentially increased heterogenicity. Inarguably, this study provided new information regarding the use of DOACs in patients with AF and complicated with AS. However, the lack of randomization renders it unpowerful to draw any definite conclusion.

The RISE MS is a pilot RCT (29) initiated in Rajaie Cardiovascular Medical and Research Center, Tehran, Iran. From May 2019 to February 2020, researchers of the study recruited 37 patients 18 to 75 years old out of a pool of 237 and they were subsequently randomized to receive either rivaroxaban 20 mg daily or warfarin (with a target international normalized ratio [INR] of 2–3) in a 1:1 ratio. Based on the inclusion criteria, the recruited patients must be diagnosed with moderate-to-severe MS and AF within the prior 12 months. The exclusion criteria excluded all the patients with high risk of bleeding, left atrial thrombi, renal impairments, or allergies to DOACs or VKA. The dosages of drugs were tightly monitored. Patients who had never been administered with anticoagulants were monitored with shorter intervals until reaching a therapeutic INR level. The primary outcomes consisted of symptomatic ischemic strokes and systemic embolic events occurred during the 12-month follow-up. TEE and brain MRI were taken at the beginning of the study, the 6th and 12th month after randomization and the results were used to evaluate thrombogenicity in the LAA and silent cerebral ischemia, respectively. There are several limitations in the study. First, the small sample size made it difficult to report robust results for primary outcomes. Furthermore, the study was discontinued for two reasons. The first reason indicated that COVID-19 was associated with higher risk of thrombotic complications. The second reason was local COVID-19 restrictions rendered a rigorous and consistent follow-up impossible. The COVID-19 restrictions also limited the patient participation in imaging examinations due to the concerns of COVID-19 contamination in the imaging center. The authors also highlighted a concern in patient enrollment. Since almost all the patients were advised with their family practitioner, sever patients with moderate to severe MS refused to participate in the study, which could become a major selection bias and confront outcome analysis. Despite the limitations, the study has generated new clinical data for the application of DOACs. The primary outcome results supported that DOACs were at least as effective as VKAs for lowering thrombotic risks in AF patients with moderate to severe MS.

With the urgency of filling the knowledge gap regarding DOACs' efficacy in treating patients with AF and MS, Zhou et al. (31) has submitted a protocol of dabigatran for stroke prevention in AF patients with moderate or severe MS (the DAVID-MS trial). According to the protocol, this will be the first open-label, multicenter, randomized clinical trial to compare the efficacy and safety of dabigatran and warfarin therapy for stroke prevention in patients with AF and moderate or severe MS. The targeted patients are those with AF aged 18 or over with moderate to severe MS without schedule for valvular intervention in the coming 12 months. Patients will be randomized in a 1:1 ratio to receive either two-doses of dabigatran (110 mg or 150 mg two times per day) or warfarin with an INR of 2–3 along with a follow-up of 12 months. The primary outcomes compose of stroke and systemic embolism and the secondary outcomes include ischaemic stroke, intracranial hemorrhage, and major bleeding. The sample size is estimated to require 686 participants and the study will be conducted mainly in Hong Kong and Mainland China. It is worth mentioning that Zhou et al. decided to use dabigatran as a comparison to warfarin not only because dabigatran appears to be more effective in stroke prevention with less intracranial bleeding than warfarin but also because of the availability of its antidote idarucizumab, granting more protection for patients involved in the DAVID-MS trial.

In summary, the guidelines for DOACs regarding its administration in AF with mitral or aortic stenosis are lacking. On the other hand, only a handful of works are done to fill in the knowledge gap. As far, there are four studies completed to explore the efficacy of DOACs in treating patients with AF and MS or AS. The two observational studies, one conducted in Korean (33) and the other one in Denmark (30), looked at the effect of DOACs in reducing thromboembolic events in patients with AF and MS or AS, respectively. The RISE-MS is a pilot RCT (29) to compare rivaroxaban to warfarin about their ability to lower risk of thromboembolism in patients with both AF and MS. DAVID-MS is a registered RCT to compare dabigatran to warfarin for the same indication above. The DAVID-MS trial (31), however, has not yet been conducted. Both the observational study by Kim et al. and the pilot RCT have reported non-inferior efficacy of DOACs compared to warfarin. The observational study conducted by Melgaard et al. (30), however, reported that DOACs are associated with higher rate of thromboembolism than warfarin. The two observational studies were subject to a variety of bias due to their retrospective nature. Therefore, their results should merely be considered as hypothesis generating but not clinically significant. The pilot RCT supported that DOACs possessed higher efficacy than warfarin, yet the study was limited to small sample size. Although DOACs have already been used widely as alternatives to traditional blood thinners such as warfarin in treatments for patients with AF, its applications in other indications like AF complicated with MS or AS have only been lightly explored. Such knowledge gap awaits elucidation as it will potentially open new windows for patients suffering from both AF and MS or AS (32).

### Future work

Although DOACs have been branded and extensively used for more than a decade, there is always ongoing research regarding their safety efficacy. A recent study conducted in Italy found that the use of DOACs is associated with higher rate of recurrent thromboembolism than VKA in patients with antiphospholipid syndrome (34). In this review, no study has included antiphospholipid syndrome in their baseline characters. Recruiting patients with the syndrome would overestimate the bleeding risk and undermine the safety outcome. Therefore, in future observational studies, researchers must consider the syndrome in baseline characteristics to avoid bias.

Another baseline characteristic that can help to optimize baseline characteristic design is VKORC genotyping (35). Patients with the VKORC gene are more susceptible to warfarin overdose, as warfarin has a narrow therapeutic window. Genetic screening on these patients can help clinicians to estimate dosages more precisely and lower the effect of VKORC polymorphism on the time required to reach targeted INR and the time required to reach stable therapeutic plasma concentration for warfarin so to lower the risk of hemorrhage (36). In the mentioned studies of our review, no information was given regarding patients' VKORC polymorphism, which could be a potential confounding factor as some patients in the warfarin arm were more likely to bleeding upon warfarin treatment (37). This could overstate the bleeding risk of warfarin compared to DOACs. Hence, we suggested that in further studies, researchers need to normalize the results along with patients' VKORC screening results.

A retrospective review conducted in Denmark reported inclusively on all-cause mortality, stroke, and bleeding in patients with AF and valvular heart disease and treated with either rivaroxaban, apixaban, or VKA (38). The goal of the study was to compare the risk of the mentioned safety event in order to infer which drug is safer. The results showed that there was non-significant absolute 2-year risk difference between VKA and DOACs groups for all outcomes measured, suggesting that apixaban and rivaroxaban possess at least equal, if not better, safety profile as VKA. Nevertheless, the limitations in this study were obvious. For instance, there was a possible detection bias that patients treated with VKA were more often in contact with practitioners and professionals and were therefore more likely to be diagnosed with arisen problems, making the VKA arm more subject to false positive detection. The other problem was that populations in the study were not stratified according to their VHD degree. This proposed a major problem in data analysis since patients with more severe VHD are more susceptible to bleeding. Therefore, if patients with different VHD severity were mixed in the same group instead of being stratified, the total bleeding events could be exaggerated or understated as there were more moderate-severe VHD patients or mild-moderate VHD patients, respectively.

### Connection to the INVICTUS trial

In the INVICTUS trial, which is the most recent RCT of DOACs, the efficacy and safety of rivaroxaban and warfarin for stroke prevention in patients who had AF due to rheumatic heart disease have been updated (39). Patients with AF and echocardiographically diagnosed rheumatic heart disease and satisfy the following criteria were enrolled: CHA2DS2VASc score of at least 2 (with higher scores suggesting a higher risk of stroke) and a mitral-valve area of no more than 2 cm^2^. In the end, there were over 80% of enrolled members in both arms with moderate-to-severe mitral stenosis. The patients were randomized in a 1:1 ratio to receive either 20 mg daily rivaroxaban or VKA. The efficacy outcomes included total stroke and systemic embolism and safety outcomes included myocardial infarction and death from vascular causes. The results showed that of 4,531 patients included in the on-treatment analysis, the occurrence rates of all stroke events of rivaroxaban and VKA groups were 1.39 and 0.87%, respectively. The rates of fatal bleeding, however, were 0.07 and 0.22 in rivaroxaban and VKA groups, respectively. In addition, VKA group also showed higher restricted mean survival time compared to rivaroxaban group, which was 1,686 days vs. 1,619 days (p = 0.002).

In connection to our review, since the INVICTUS enrolled mostly MS patients with rheumatic heart disease and AF, we can make inference accordingly. Compared to the observational studies in our review, the data from INVICTUS supported otherwise opposite conclusion as the INVICTUS have suggested that for preventing thromboembolic events in rheumatoid heart disease patients with AF, VKA is associated with better efficacy and lower mortality rate compared to rivaroxaban, although with higher bleeding rate. However, the authors of INVICTUS indicated that there was no relation between AF-related stroke prevention and reduced mortality rate. VKA also did not slow down the deterioration of heart-valve, which suggested that the better efficacy in preventing stroke and lower mortality in the VKA group was not related to MS progression. On the other hand, although the rivaroxaban group had higher mortality rate, there was no evidence to suggest rivaroxaban increased mortality among the patients, as it has been shown that rivaroxaban lowers mortality substantially in patients with atherosclerotic vascular disease (40). Hence, if VKA did not lower mortality through optimizing AF-related stroke prevention or slowing MS progression, it appeared more likely that VKA had a direct effect on the disease process of rheumatic heart disease. This information is important because if the efficacy of stroke prevention of VKA or DOACs is dependent on rheumatic heart disease progression, such condition should strictly be included as one of the exclusion criteria when studying the effectiveness of stroke prevention of DOACs vs. VKA in patients with AF and MS.

## Conclusions

Among the reviewed studies (29–31, 33), two of them showed non-inferiority of DOACs to warfarin in treating patients with AF and mitral or aortic stenosis, and one observational study showed the opposite results. Due to their own limitations, the use of DOACs in AF patients with MS or AS is still controversial. A more adequately designed RCT with a larger sample size is needed to verify the results from the previous studies. Warfarin would remain the drug of choice for such patients as per the guideline, due to the lack of clinical data, until a more definitive trial showed otherwise.

## Data availability statement

The original contributions presented in the study are included in the article/supplementary material, further inquiries can be directed to the corresponding authors.

## Author contributions

All authors listed have made a substantial, direct, and intellectual contribution to the work and approved it for publication.

## Conflict of interest

The authors declare that the research was conducted in the absence of any commercial or financial relationships that could be construed as a potential conflict of interest.

## Publisher's note

All claims expressed in this article are solely those of the authors and do not necessarily represent those of their affiliated organizations, or those of the publisher, the editors and the reviewers. Any product that may be evaluated in this article, or claim that may be made by its manufacturer, is not guaranteed or endorsed by the publisher.
